# DCAF-Net: Density-Conditioned Attention Fusion Network for Single-Image Dehazing

**DOI:** 10.3390/s26144656

**Published:** 2026-07-22

**Authors:** Nianfeng Li, Shaojie Liu, Hongjie Ding, Shenyan Gao, Zhiguo Xiao, Qian Liu

**Affiliations:** 1 College of Computer Science and Technology, Changchun University, Changchun 130022, China; 241501505@mails.ccu.edu.cn (S.L.); 241502529@mails.ccu.edu.cn (H.D.); 241503563@mails.ccu.edu.cn (S.G.); 3220215169@bit.edu.cn (Z.X.); liuq@ccu.edu.cn (Q.L.); 2 School of Computer Science & Technology, Beijing Institute of Technology, Beijing 100811, China

**Keywords:** image dehazing, non-homogeneous fog distribution, multi-prior fusion, density-conditioned attention

## Abstract

Single-image dehazing aims to recover clear scenes from degraded images affected by atmospheric scattering, serving as a critical preprocessing technique for improving the imaging quality of visual sensors. Existing deep learning-based dehazing methods exhibit limited generalization ability in real-world scenarios, primarily due to the spatial non-uniformity of haze and its coupling with illumination and texture degradation, as well as the scarcity of real paired data. To address these issues, this paper proposes a haze-density conditional attention fusion network (DCAF-Net). The network employs an adaptive haze density perception module to fuse priors such as the dark channel, local contrast, and saturation, generating a spatial haze density guidance map. This map is then embedded as conditional information into the multi-scale feature modulation and attention fusion process, enabling adaptive restoration of regions with different degradation levels. Furthermore, a residual dense cascaded feature enhancement module is designed to leverage feature reuse, gated fusion, and residual learning to enhance the representational capacity of deep features. Training adopts a joint optimization objective combining Charbonnier reconstruction loss, perceptual contrast loss, and structural similarity loss. Experimental results demonstrate that DCAF-Net achieves competitive performance against representative methods on multiple synthetic and real-world hazy datasets, and shows promising restoration performance on representative real-world hazy scenes, and can provide high-quality image preprocessing support for visual-sensor-based intelligent perception systems.

## 1. Introduction

Single-image dehazing aims to recover scene content with clear structures, natural colors, and high visual quality from images degraded by atmospheric factors such as fog and haze. Haze is typically caused by the absorption and scattering of light by suspended particles in the atmosphere. It markedly reduces image contrast, weakens texture details, and introduces color shifts, thereby impairing visual perception and the reliability of downstream high-level vision tasks. In applications like autonomous driving, remote sensing, intelligent surveillance, and mobile imaging, haze degradation can degrade the performance of object recognition, scene understanding, and decision-making systems. Consequently, single-image dehazing has drawn extensive attention from both academia and industry over the past decade [1].

Early single-image dehazing methods mainly relied on the atmospheric scattering model and handcrafted priors, such as the dark channel prior [2], color attenuation prior [3], contrast-based constraints [4], and non-local color statistics [5]. These methods estimate the transmission map and atmospheric light to recover a clear image and therefore offer physical interpretability. However, their effectiveness is often limited by the validity of the underlying assumptions, particularly in scenes containing large sky regions, strong illumination, bright objects, spatially non-uniform haze, or mixed degradations. With the availability of paired training data, deep learning-based methods have shifted the focus from explicit parameter estimation to end-to-end image restoration. CNN-based [6], multi-scale [7], attention-based [8], and Transformer-based networks have achieved substantial progress on synthetic benchmarks [9,10]. Nevertheless, many of these methods model haze degradation implicitly through learned features, which may be insufficient for adapting feature responses to large spatial variations in real-world haze.

Despite this progress, robust dehazing in real-world scenes remains challenging. Most supervised methods are trained predominantly on synthetic hazy/clear image pairs, whereas real haze is affected by scene depth, aerosol distribution, illumination, camera response, sensor noise, and potentially coupled weather degradations. Consequently, the haze distributions and image statistics of synthetic and real data may differ substantially. This synthetic-to-real domain gap can lead to inconsistent restoration across regions, residual haze in heavily degraded areas, and color distortion under complex illumination. Although real-world paired datasets, such as O-HAZE and NH-HAZE, provide valuable evaluation benchmarks, their limited scale and diversity cannot fully cover the variability of real atmospheric conditions [11]. Therefore, improving adaptation to spatially varying haze while maintaining reliable feature representation remains an important challenge for single-image dehazing.

To address these challenges, we investigate single-image dehazing from the complementary perspectives of spatial degradation-aware feature modulation and deep feature representation enhancement. Existing attention-based dehazing networks can selectively enhance informative features, and dense or residual connections can improve feature propagation. However, attention weights are often inferred implicitly from learned features without an explicit spatial cue describing haze-related degradation. In addition, conventional residual or dense designs do not necessarily distinguish between the current feature response and reusable representations from preceding stages. Motivated by these observations, we propose a Density-Conditioned Attention Fusion Network (DCAF-Net). DCAF-Net uses complementary haze-related priors to construct a learnable spatial guidance signal and employs gated dense feature reuse at the bottleneck stage. The former encourages degradation-aware feature modulation across scales, while the latter improves the propagation and utilization of deep contextual information for severely degraded regions.

Specifically, we design an adaptive Haze Density-aware Module (HDM) that integrates the dark channel, local contrast, and saturation as complementary haze-related cues. Rather than estimating the exact physical haze density, HDM generates a haze density guidance map that provides spatially varying degradation cues for multi-scale feature modulation and attention fusion. This design enables the network to adapt its feature responses to regions with different degradation characteristics. In addition, we propose a residual Dense Feature Enhancement module (DFE) at the bottleneck stage. By combining cross-layer feature reuse, gated fusion, and residual propagation, DFE promotes effective information flow and feature utilization, thereby strengthening structural and texture recovery in heavily degraded regions. HDM and DFE are integrated into a unified encoder–decoder framework and optimized end-to-end with a joint objective that balances pixel fidelity, structural consistency, and local contrast restoration.

The main contributions of this paper are as follows:

1. We propose a Density-Conditioned Attention Fusion Network (DCAF-Net) to enhance the adaptability of single-image dehazing models to non-uniform haze and complex real-world degradation.
2. We design an adaptive Haze Density-aware Module (HDM) that fuses complementary priors, including dark channel, local contrast, and saturation, to generate a spatial haze density guidance map. This map is utilized for multi-scale feature modulation and attention fusion, thereby improving the model’s ability to capture spatial variations in haze distribution.
3. We propose a residual Dense Feature Enhancement module (DFE), which improves information propagation and utilization efficiency of deep features through feature reuse, gated fusion, and residual propagation. This strengthens the model’s representational capacity for heavily hazy regions and areas with local detail degradation.
4. We conduct systematic experiments on multiple synthetic datasets and real-world hazy images. Results demonstrate that the proposed DCAF-Net achieves competitive performance in both quantitative metrics and visual quality, and shows promising restoration capability on representative real-world non-uniform hazy scenes.

The remainder of this paper is organized as follows. Section 2 reviews traditional single-image dehazing methods, deep learning-based dehazing methods, and prior-guided approaches. Section 3 details the proposed DCAF-Net, including the overall network architecture, HDM, DFE, and the loss function design. Section 4 presents the experimental setup, quantitative and qualitative comparison results, and ablation analysis. Section 5 concludes the paper and discusses limitations and future research directions.

## 2. Related Work

In single-image dehazing, existing technical approaches can be broadly divided into two categories: prior-based dehazing methods and learning-based dehazing methods. Prior-based methods model the statistical regularities or physical properties involved in the degradation of hazy images. They introduce explicit priors to constrain the solution space and reconstruct clear images accordingly. These priors include the dark channel prior, color-line prior, contrast statistics, and physical imaging constraints. In contrast, learning-based methods rely on supervised or weakly supervised training frameworks. They achieve end-to-end restoration through convolutional networks, attention mechanisms, and multi-scale feature modeling.

### 2.1. Prior-Based Dehazing Methods

Prior-based single-image dehazing methods are built on the atmospheric scattering model. Their core idea is to estimate atmospheric light and transmission from a single observed image by imposing manually designed statistical or physical constraints, and then recover the clear image through inversion. In this line of research, Tan improved visibility by enhancing local contrast and highlighted haze-induced contrast attenuation as a central phenomenon [12]. Fattal decoupled scene reflectance from medium transmission based on statistical independence, providing a feasible framework for subsequent parametric estimation [13]. He, Sun, and Tang proposed the dark channel prior. This method estimates transmission by exploiting the local minimum channel distribution in haze-free natural images, and improves edge quality through refinement strategies such as soft matting. It has become a representative baseline among prior-based methods [2]. To improve computational efficiency, He et al. further introduced guided filtering to accelerate transmission refinement, making this class of methods more practical [14]. Tarel and Hautière emphasized fast visibility restoration and achieved a balance between real-time performance and visual quality [15]. Meng introduced contextual regularization on top of boundary constraints, which alleviated transmission discontinuities in regions with abrupt depth changes [16]. Zhu, Mai, and Shao proposed the color attenuation prior, which uses the relationship between brightness and saturation to provide depth cues and reduces reliance on the single dark channel assumption [17]. Berman and Avidan developed the Haze-lines model based on non-local color clustering, improving the stability of parameter estimation from the perspective of global color structure [18].

Overall, prior-based methods provide clear physical interpretability and require little or no paired training data. However, their performance strongly depends on whether the assumed priors hold in the observed scene. In cases involving large sky regions, strong light sources, highly reflective objects, non-uniform haze, or mixed atmospheric degradation, these methods may produce residual haze, halo artifacts, over-enhancement, or color distortion. Moreover, most prior-based approaches rely on multi-stage estimation and parameter-sensitive post-processing, which limits their robustness across diverse real-world scenes. Nevertheless, these priors reveal useful haze-related image statistics. Instead of directly using fixed priors for model inversion, recent learning-based methods increasingly incorporate such priors as auxiliary cues to guide feature representation and restoration.

### 2.2. Learning-Based Dehazing Methods

In recent years, single-image dehazing methods based on deep learning have gradually become the mainstream research direction in this field because of their strong capabilities in feature extraction and pattern recognition. Early deep learning models mainly focused on accurately estimating the key physical parameters in the atmospheric scattering model through convolutional neural networks (CNNs). For example, the classic DehazeNet (Cai et al., 2016) used multi-scale mapping and Maxout units to extract features and estimate the transmission map in an end-to-end manner [6]. To overcome the limitations of estimating only a single parameter, Cascaded CNN (Li et al., 2018) adopted a cascaded architecture and achieved joint estimation of the transmission map and atmospheric light by sharing hidden-layer features [19]. Aggregated Resolution CNN (He et al., 2019) reconstructed a more robust transmission map by integrating multi-resolution downsampled features and combining them with residual mapping [20]. To improve efficient feature fusion, FAMED-Net (Zhang et al., 2020) achieved fast multi-scale dehazing through pointwise convolution and an encoder fusion strategy [21].

To avoid the accumulated errors caused by stage-wise estimation of physical parameters, later studies gradually shifted toward end-to-end direct restoration models. AOD-Net (Li et al., 2017) unified the transmission map and atmospheric light into a single reformulated parameter and directly reconstructed a clear image from the hazy input [22]. On this basis, network design increasingly emphasized multi-scale feature aggregation and attention mechanisms. GridDehazeNet (Liu et al., 2019) proposed an attention-guided multi-scale grid architecture, which effectively alleviated the bottleneck in cross-scale information exchange [23]. More recently, Transformer architectures have been successfully introduced into the dehazing field. For example, DeHamer, proposed by Guo et al. (2022), achieved excellent performance in non-uniform haze scenes by using transmission-aware 3D positional embeddings [24]. Dong et al. (2024) further combined deformable convolution with Transformers and developed DehazeDCT, which improved computational efficiency and feature aggregation for high-resolution images [25]. At the same time, increasing attention has been paid to detail enhancement and the use of frequency-domain priors. DEA-Net (Chen et al., 2024) introduced detail-enhanced convolution and a content-guided attention mechanism to better preserve high-frequency structures [26]. Zhang et al. moved beyond the spatial domain and exploited frequency-domain priors through cross-domain Fourier convolution, significantly improving restoration quality [27].

To reduce the reliance on paired training data, generative adversarial networks (GANs), unsupervised learning, and domain-adaptive frameworks have been investigated. Cycle-Dehaze (Engin et al., 2018) achieved unpaired image dehazing by combining cycle consistency with a Laplacian pyramid to preserve edge structures [28]. Liu et al. (2020) designed a two-stage cyclic mapping framework with both dehazing and re-hazing subnetworks, while incorporating sky prior knowledge to improve the naturalness of generated images [29]. More recent studies have explored feature disentanglement, contrastive regularization, and cross-domain transfer to improve the adaptability of unsupervised models to real-world hazy scenes [30]. However, these methods may still be affected by training instability, domain-specific assumptions, and the lack of reliable full-reference supervision in real scenes.

Prior-guided deep dehazing has also received increasing attention. Instead of using handcrafted priors as fixed constraints for direct model inversion, these methods embed haze-related priors into learnable networks as auxiliary information. Dark channel, color attenuation, contrast statistics, transmission-related cues, frequency-domain priors, and multi-level prior information have been used to guide feature extraction, domain adaptation, or reconstruction [31,32]. However, existing prior-guided methods often use priors as input augmentation, auxiliary regularization, hierarchical coordination cues, or domain-alignment constraints. In contrast, the proposed HDM fuses dark channel, local contrast, and saturation to generate a spatial haze density guidance map for multi-scale feature modulation and attention fusion, thereby incorporating prior information as an adaptive degradation-aware modulation signal.

Although learning-based dehazing methods have achieved substantial progress, robust restoration in complex real-world scenes remains challenging. First, many supervised models are trained mainly on synthetic paired data, and their performance may degrade when haze distribution, illumination, noise, or color statistics differ from the training domain. Second, attention-based or multi-scale networks usually learn degradation responses implicitly, which may be insufficient for explicitly adapting restoration to spatially varying haze. Third, repeated feature transformation in deep networks may weaken the propagation and reuse of useful contextual information, particularly in heavily degraded regions. These observations motivate our DCAF-Net, which combines haze density guidance based on complementary priors with residual dense feature enhancement to improve adaptive restoration under non-uniform haze and complex degradation.

## 3. Proposed Model

### 3.1. Overall Network Architecture

This paper develops a hierarchical encoder-decoder network for single-image dehazing. The network takes a hazy image I as input and first performs initial feature mapping through a shallow convolutional layer. It then progressively extracts feature representations at different scales through two downsampling stages. After deep feature enhancement at the bottleneck layer, the network gradually restores the spatial resolution through two upsampling stages and finally outputs the clear image $\hat{J}$. Meanwhile, to strengthen cross-scale information interaction, a content-guided feature fusion mechanism is introduced between the encoder and decoder. In addition, to handle non-uniform haze distribution and improve deep feature representation, this paper further incorporates two key modules, namely HDM and DFE.

**Figure 1:**
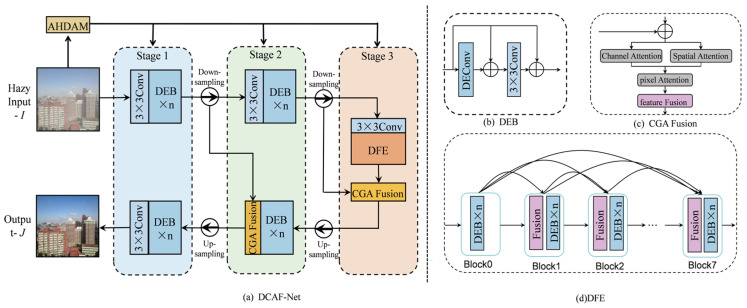
Overall architecture of the proposed dehazing network, DCAF-Net.

As shown in Figure 1, the overall network consists of three encoding stages, one deep enhancement stage, and two decoding and reconstruction stages. Stage 1 mainly extracts shallow texture and edge information. Stage 2 is used to model intermediate-scale structural representations. Stage 3 further learns deeper and more semantic degradation features. In the deep stage, the proposed method replaces a simple sequential enhancement structure with the DFE module to strengthen historical feature reuse and cascaded modeling capability. Meanwhile, HDM serves as an additional perception branch. It estimates a spatial haze density map from the input image and uses it as conditional information to modulate feature flows at multiple scales, thereby improving the network’s ability to perceive spatially varying haze distributions.

### 3.2. Adaptive Haze Density Awareness Module

In real-world scenes, haze degradation is usually not uniformly distributed. The degree of visibility degradation may vary significantly across different regions due to changes in scene depth, lighting conditions, and atmospheric scattering intensity. If the network applies the same feature processing strategy to all spatial locations, it may fail to produce sufficiently discriminative responses between heavily hazy regions and lightly hazy regions. To address this issue, this paper designs an Adaptive Haze Density Awareness Module (HDM). This module estimates a spatial haze density map from the input hazy image and uses it as a conditional signal to guide multi-scale features.

**Figure 2:**
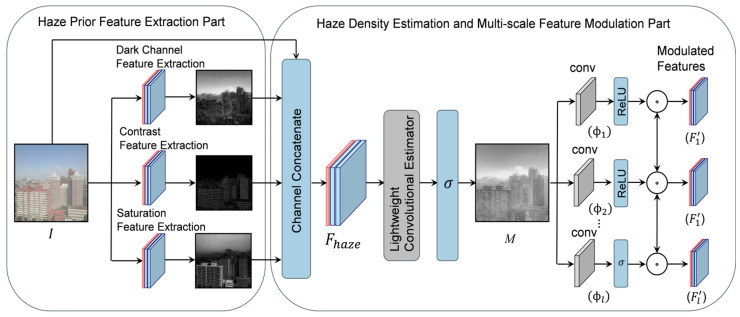
Detailed architecture of the Adaptive Haze Density Awareness Module (HDM).

As shown in Figure 2, HDM mainly consists of two parts: a haze-prior feature extraction component and a haze density estimation component. Specifically, the module first extracts dark channel features, local contrast features, and saturation features from the input image. These priors, together with the original RGB image, are then fed into a lightweight convolutional estimator to generate a single-channel haze density map. The resulting density map is subsequently projected into feature spaces at different scales and used to condition the encoded features.

For the input image I, the dark channel feature can be expressed as follows:

(1) $$\begin{array}{c} D\left(x\right)=\underset{y\in \Omega \left(x\right)}{\min}\left(\underset{c\in r,g,b}{\min}I^{c}\left(y\right)\right) \end{array}$$

here, Ω(x) denotes the local neighborhood centered at pixel location x, and $I^{c}(y)$ represents the response at pixel location y in the c-th color channel. In dehazing, the dark channel prior is generally regarded as an informative statistical feature. Therefore, it can serve as an auxiliary cue for haze density estimation.

To characterize the degree of local structural degradation, this paper further introduces contrast features. Let G denote the grayscale image. Then the local contrast response can be defined as follows:

(2) $$\begin{array}{c} C\left(x\right)=\left|\Delta G\left(x\right)\right| \end{array}$$

here, Δ denotes the discrete Laplacian operator. Because haze typically weakens edge and texture information, this feature can reflect local visibility changes to a certain extent.

In addition, to describe the color attenuation effect, this paper also introduces saturation features. Let the mean channel value of the input image at location x be defined as follows:

(3) $$\begin{array}{c} \mu \left(x\right)=\frac{1}{3}\sum_{c\in r,g,b}I^{c}\left(x\right) \end{array}$$

Then, its saturation can be expressed as follows:

(4) $$\begin{array}{c} S\left(x\right)=\sqrt{\frac{1}{3}\sum_{c\in r,g,b}{\left(I^{c}\left(x\right)-\mu \left(x\right)\right)}^{2}+\varepsilon } \end{array}$$

here, ε is a small constant introduced to ensure numerical stability.

The dark channel, local contrast, and saturation priors are selected because they describe haze degradation from complementary perspectives. The dark channel prior is closely related to haze accumulation and transmission attenuation, and can provide useful cues for dense haze regions. Local contrast is considered because atmospheric scattering reduces image contrast and weakens structural details, especially in low-visibility regions. Saturation is introduced because haze often causes color attenuation and reduces color vividness. Therefore, these three priors jointly provide intensity-related, structure-related, and color-related degradation cues for generating the haze density guidance map. It should be noted that this map is used as a degradation-aware guidance signal rather than an exact physical haze density estimation, since existing dehazing datasets generally do not provide pixel-level haze density annotations or atmospheric scattering coefficient maps.

After obtaining the above prior features, this paper concatenates the original image and its prior features along the channel dimension to construct the input for haze density estimation:

(5) $$\begin{array}{c} F_{\mathrm{haze}}=\mathrm{Cat}\left(I,D,C,S\right) \end{array}$$

The concatenated features are modeled by a convolutional estimator, and a normalized haze density map is produced through a Sigmoid activation function:

(6) $$\begin{array}{c} M=\sigma \left(H\left(F_{\mathrm{haze}}\right)\right) \end{array}$$

here, H(⋅) denotes the haze density estimation network, and σ(⋅) is the Sigmoid function. The resulting $M\in [0,1{]}^{H\times W}$ can be regarded as a spatial haze density response map of the input image, where larger values usually correspond to regions with relatively heavier haze.

To enable this density information to act on features at different scales, this paper interpolates the density map M to match the spatial size of each feature map and then projects it to the corresponding channel dimension through convolution. Let the feature at the l-th scale be $F_{l}\in R^{C_{l}\times H_{l}\times W_{l}}$, and let the resized density map at the same scale be denoted by ${\tilde{M}}_{l}$. Then the modulation weight can be expressed as follows:

(7) $$\begin{array}{c} W_{l}=\sigma \left(\phi_{l}\left(\tilde{M_{l}}\right)\right) \end{array}$$

here, $\phi_{l}(\cdot )$ denotes the scale-specific linear projection mapping. Using this modulation weight to perform element-wise modulation on the feature map yields:

(8) $$\begin{array}{c} F_{l}^{\prime }=F_{l}⊙W_{l} \end{array}$$

here, ⊙ denotes element-wise multiplication. In this way, HDM explicitly incorporates haze distribution information from the input image into multi-scale feature representations, enabling the network to produce more targeted responses in different spatial regions. Compared with fixed global enhancement strategies, this module functions more like a flexible condition-aware mechanism and can significantly improve dehazing performance in non-uniform haze scenes.

It should be noted that HDM differs from general attention modules that infer feature importance only from learned representations. By integrating complementary haze-related priors, HDM provides an explicit degradation-aware spatial cue for adaptive feature modulation. Unlike traditional prior-based methods that directly use handcrafted priors for atmospheric model inversion, HDM embeds these priors into a learnable network as auxiliary guidance. Therefore, the generated haze density guidance map is used to guide region-adaptive feature responses rather than to represent an exact physical haze density.

### 3.3. Residual Dense Cascaded Feature Enhancement Module

In an encoder-decoder architecture, bottleneck features usually possess a large receptive field and strong semantic abstraction capability. Therefore, their representation quality has an important influence on the subsequent decoding and restoration process. However, if deep features are processed only by simple sequential enhancement blocks, the intermediate representations generated at different stages may not be fully reused. In addition, long sequential propagation may make the feature update process overly homogeneous. To address this issue, this paper proposes a Deep Feature Enhancement (DFE) module to perform more effective cascaded enhancement on deep features.

As shown in Figure 1, DFE is placed in Stage 3 of the network, namely the deep enhancement stage. Its core idea is to reuse historical features by aggregating the outputs of previous stages, and to adaptively balance the current input features and the aggregated historical features through a gating mechanism. A global residual connection is then introduced to obtain more stable deep feature enhancement.

Let the input feature of DFE be denoted by $F_{0}$. The module consists of N enhancement stages. The first stage directly enhances the input feature and can be expressed as follows:

(9) $$\begin{array}{c} F_{1}=B_{1}\left(F_{0}\right) \end{array}$$

here, $B_{1}(\cdot )$ denotes the first enhancement unit.

For the i-th stage $\left(i>1\right)$, the outputs of all preceding stages are first concatenated along the channel dimension:

(10) $$\begin{array}{c} F_{i}^{\mathrm{cat}}=\mathrm{Cat}\left(F_{1},F_{2},\ldots ,F_{i-1}\right) \end{array}$$

Direct concatenation may introduce substantial channel redundancy. Therefore, this paper further applies a feature aggregation mapping $A_{i}(\cdot )$ to compress and refine the concatenated features, yielding the aggregated historical feature:

(11) $$\begin{array}{c} F_{i}^{\mathrm{agg}}=A_{i}\left(F_{i}^{\mathrm{cat}}\right) \end{array}$$

here, $A_{i}(\cdot )$ consists of a channel compression operation and a channel attention mapping, which are used to preserve useful historical information while suppressing redundant responses.

After obtaining the aggregated historical feature, this paper adopts a gated fusion mechanism to adaptively combine the current feature stream with the historical feature stream. Let the input of the main branch at the current stage be $F_{i-1}$. Then the gating weight is defined as follows:

(12) $$\begin{array}{c} G_{i}=\sigma \left(Gi\left(\mathrm{Cat}\left(F_{i-1},F_{i}^{\mathrm{agg}}\right)\right)\right) \end{array}$$

here, $G_{i}(\cdot )$ denotes the gating mapping network, and σ(⋅) is the Sigmoid function. Accordingly, the fused input to the current stage can be expressed as follows:

(13) $$\begin{array}{c} \hat{F_{i}}=G_{i}⊙F_{i-1}+\left(1-G_{i}\right)⊙F_{i}^{\mathrm{agg}} \end{array}$$

The fused result is then fed into the enhancement unit of the current stage to obtain the corresponding stage output:

(14) $$\begin{array}{c} F_{i}=B_{i}\left(\hat{F_{i}}\right),\;\;i=2,3,\ldots ,N \end{array}$$

here, $B_{i}(\cdot )$ denotes the i-th enhancement unit. In the implementation of this paper, this unit is designed to further improve the representation of local details and structural information.

To prevent deep features from deviating excessively from the original representation during multi-stage enhancement, DFE introduces a global residual connection at the outer level of the module. The final output can be written as follows:

(15) $$\begin{array}{c} F_{\mathrm{out}}=F_{N}+F_{0} \end{array}$$

This design preserves a direct transmission path for the input feature in a formal sense. It may help improve optimization stability and mitigate potential information loss during deep feature enhancement.

Overall, DFE can be viewed as a progressive deep modeling strategy that follows three steps: historical feature aggregation, gated fusion, and current-stage enhancement. Compared with a simple sequential stacking structure, this module is structurally more conducive to the repeated use of intermediate features. At the same time, the gating mechanism helps suppress redundant accumulation to some extent. For the dehazing task, this more comprehensive bottleneck feature modeling strategy is expected to provide more discriminative feature support for subsequent decoding and image reconstruction.

Compared with standard residual or dense blocks, DFE not only reuses preceding features but also introduces gated fusion to adaptively balance the current feature response and aggregated historical representations. The residual propagation further facilitates stable information flow at the bottleneck stage. This design improves deep feature utilization and enhances the representation of heavily degraded regions, where structural information and texture details are often difficult to recover.

### 3.4. Composite Loss Function Design

**Figure 3:**
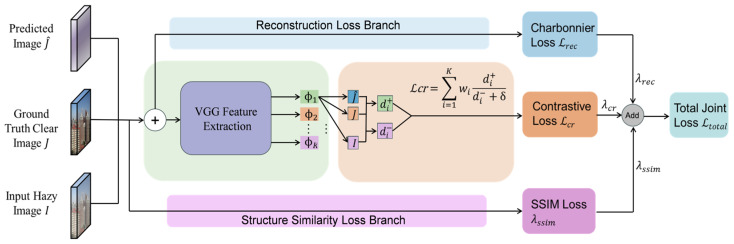
Composite Loss Function.

To jointly enforce pixel-level fidelity, perceptual consistency, and structural similarity in the restored results, this paper adopts a unified optimization objective composed of reconstruction loss, contrastive loss, and structural similarity loss, as shown in Figure 3. The overall loss function is defined as follows:

(16) $$\begin{array}{c} L_{total}=\lambda_{rec}L_{rec}+\lambda_{cr}L_{cr}+\lambda_{ssim}L_{\mathrm{ssim}} \end{array}$$

here, $\lambda_{rec}$, $\lambda_{cr}$, and $\lambda_{ssim}$ denote the weighting coefficients for the reconstruction loss, contrastive loss, and structural similarity loss, respectively.

First, in the pixel space, this paper adopts the Charbonnier loss as the primary reconstruction constraint. This loss can be regarded as a smooth approximation of the $L_{1}$ loss, and it is defined as follows:

(17) $$\begin{array}{c} Lrec=\frac{1}{N}\sum p\sqrt{{\left(\hat{J_{p}}-J_{p}\right)}^{2}+\epsilon^{2}} \end{array}$$

here, p denotes the pixel location, N is the total number of pixels in the image, and ϵ is a small constant. Compared with the direct use of an $L_{2}$-form loss, this loss usually provides smoother gradient behavior during training and may be more robust to outlier errors.

Second, to enhance the consistency between the restored result and the ground-truth clear image in the high-level perceptual space, while maintaining an appropriate distinction from the input hazy image, this paper introduces a contrastive loss based on the feature space of a pretrained VGG network. Let $\phi_{i}(\cdot )$ denote the feature extraction mapping at the i-th perceptual layer. Then the positive distance between the output image and the ground-truth image, and the negative distance between the output image and the input hazy image, are defined as follows:

(18) $$\begin{array}{c} d_{i}^{+}={\left|\phi_{i}\left(\hat{J}\right)-\phi_{i}\left(J\right)\right|}_{1} \end{array}$$

(19) $$\begin{array}{c} d_{i}^{-}={\left|\phi_{i}\left(\hat{J}\right)-\phi_{i}\left(I\right)\right|}_{1} \end{array}$$

Accordingly, the contrastive loss can be written as follows:

(20) $$\begin{array}{c} Lcr=\sum_{i=1}^{K}w_{i}\frac{d_{i}^{+}}{d_{i}^{-}+\delta } \end{array}$$

here, $w_{i}$ denotes the weight associated with the i-th feature layer, and δ is a constant introduced to ensure numerical stability. In form, this loss encourages the restored result to be closer to the clear image in the perceptual space while remaining relatively farther from the input hazy image.

In addition, to further constrain the consistency between the restored result and the ground-truth image in terms of luminance, contrast, and local structure, this paper introduces a structural similarity loss, which is defined as follows:

(21) $$\begin{array}{c} L_{\mathrm{ssim}}=1-\mathrm{SSIM}\left(\hat{J},J\right) \end{array}$$

here, SSIM(⋅,⋅) denotes the structural similarity index. This term provides supplementary structural supervision for the restored result and can improve local texture quality and overall visual perception to a certain extent.

Overall, the proposed joint loss constrains the restored result from three complementary perspectives: pixel-level fidelity, feature-level perceptual representation, and structural consistency. The Charbonnier reconstruction loss provides robust pixel-wise supervision and encourages the restored image to be close to the ground truth in the image domain. The contrastive loss guides the restored image features to approach the clear image features while moving away from the hazy image features in the perceptual feature space, thereby enhancing feature-level discriminability. The SSIM loss further provides structural similarity constraints to help preserve local image structures. By combining these three terms, the dehazing network receives a more comprehensive optimization signal than using only pixel-wise reconstruction loss, which helps balance objective evaluation metrics and subjective visual quality.

## 4. Experiments and Analysis

This chapter systematically evaluates the effectiveness and generalization ability of the proposed dehazing method through extensive experiments. It first introduces the experimental setup, including the implementation platform, training strategy, loss function configuration, and data preprocessing procedure. The proposed method is then quantitatively and qualitatively compared with representative existing dehazing methods on synthetic datasets and public test benchmarks, in order to comprehensively assess its performance in structural restoration, detail preservation, and overall visual naturalness. In addition, ablation studies are conducted to analyze the effectiveness of HDM, DFE, and the joint loss function. The experimental results show that the proposed network can model spatially non-uniform haze degradation more effectively and achieves strong overall performance in restoring image clarity, texture details, and structural consistency.

### 4.1. Implementation Details

The proposed DCAF-Net is implemented in PyTorch 2.4.1, and all experiments are conducted on a workstation equipped with an NVIDIA GeForce RTX 4090 GPU with 24 GB memory. The network is optimized using the Adam optimizer, with momentum parameters set to $\beta_{1}=0.9$ and $\beta_{2}=0.999$, and the numerical stability term set to $1\times 10^{-8}$. The batch size is set to 4, and the network is trained for 100 epochs, with each epoch containing 5000 iterations. The initial learning rate is set to $4\times 10^{-4}$ and is gradually reduced to $1\times 10^{-6}$ using a cosine decay schedule to improve convergence stability in the later training stage. All experiments are trained from scratch without relying on any additional large-scale pretrained models.

To ensure reproducibility, the random seed is fixed throughout training. During training, hazy images and their corresponding clear images are paired and loaded according to filename matching, and online data augmentation is adopted to increase training diversity and reduce the risk of overfitting. Specifically, the input images are randomly cropped into patches of 256×256 and randomly rotated by 0°, 90°, 180°, or 270°. No random augmentation is used during testing, and inference is performed directly on the full image. For input images whose sizes do not satisfy the downsampling requirements of the network, boundary padding is applied first, and the output is then cropped back to the original size for evaluation after inference.

### 4.2. Datasets and Evaluation Metrics

The proposed DCAF-Net is trained and evaluated on synthetic uniform haze datasets, real-world uniform haze datasets, and non-uniform haze datasets. The datasets used in this study include ITS, OTS, O-Haze [33], and NH-Haze [34]. Among them, ITS and OTS are derived from the RESIDE benchmark [35] and are used to evaluate indoor and outdoor synthetic uniform haze scenes, respectively. ITS contains 110,500 indoor synthetic hazy images. For indoor experiments, the model is trained on the ITS dataset and tested on the 500 images in SOTS-Indoor, with all image resolutions uniformly resized to 640×480. OTS contains 313,950 outdoor synthetic hazy images. For outdoor experiments, the model is trained on the OTS dataset and tested on the 500 images in SOTS-Outdoor, with the image resolution similarly adjusted to 1600×1200.

O-Haze is a real-world uniform haze dataset consisting of 45 pairs of hazy and corresponding clear images captured in outdoor environments using haze machines. Among them, 35 pairs are used for training, 5 for validation, and 5 for testing. Because the original image sizes are not uniform, all images are resized to 1600 × 1200 in the experiments. Considering the limited size of the official test split, we additionally conduct 5-fold cross-validation on the O-Haze dataset and report the average performance over the five folds to reduce the influence of a specific data partition. NH-Haze is a non-uniform haze dataset containing 55 pairs of hazy and clear images, all with an original resolution of 1600 × 1200. It is divided into 45 training images, 5 for validation, and 5 for testing. The distribution of these datasets is summarized in Table 1.

During model evaluation, the Structural Similarity Index Measure (SSIM) [36] and Peak Signal-to-Noise Ratio (PSNR) are adopted as the primary objective evaluation metrics. PSNR is used to measure the pixel-level error between the reconstructed image and the reference image, while SSIM evaluates structural similarity and visual consistency. In addition, this paper combines subjective visual comparisons to further assess the performance of different methods in terms of residual haze suppression, color restoration, texture detail recovery, and overall visual naturalness.

### 4.3. Comparison with Representative Dehazing Methods

To validate the effectiveness of the proposed method, this paper compares it with traditional prior-based methods and representative deep learning-based methods on multiple public benchmark datasets. For methods with publicly available pretrained models, the official models are directly used for testing. For methods without released weights, the models are retrained on the corresponding training sets based on the public code, while following the original parameter settings as closely as possible. All methods are evaluated under the same test conditions, and PSNR and SSIM are used to measure reconstruction accuracy and structural fidelity, respectively.

#### 4.3.1. Quantitative Comparison

Table 2 presents the quantitative results of different methods on SOTS-Outdoor, SOTS-Indoor, NH-HAZE, and O-HAZE. The first two datasets are mainly used to evaluate dehazing performance in synthetic uniform haze scenes, whereas the latter two are used to assess generalization under real-world haze and non-uniform haze conditions.

Overall, the traditional prior-based method DCP and the early shallow network DehazeNet perform noticeably worse than deep learning-based methods. This finding suggests that fixed priors or shallow mappings are insufficient to fully model complex haze degradation. In recent years, deep models such as GridDehazeNet, MSBDN, FFA-Net, DeHamer, DEA-Net, JSFC-Net, and DNMGDT have achieved substantial improvements on multiple benchmarks. Their success further demonstrates the effectiveness of deep feature learning for image dehazing through multi-scale feature modeling, attention enhancement, and context aggregation.

As shown in Table 2, the proposed method delivers stable overall performance across all four test benchmarks and shows a more pronounced advantage on real-world haze datasets. On NH-HAZE, the proposed method achieves a PSNR of 21.57 dB and an SSIM of 0.815, both of which are higher than those of the other competing methods. This result indicates that the proposed model can effectively handle real-world haze degradation with spatially non-uniform distributions. On O-HAZE, the proposed method attains the best PSNR of 25.86 dB and an SSIM of 0.823, indicating strong capability in preserving structure and recovering details in real-world uniform haze scenes. Although PGH^2^Net achieves the best SSIM due to its multi-prior-guided structure restoration design, its PSNR remains less competitive because it cannot perfectly recover the colors of real haze scenes, making it less favorable under a pixel-error-sensitive metric.

Compared with the strong baseline JSFC-Net, the proposed method improves the PSNR/SSIM from 21.42/0.799 to 21.57/0.815 on NH-HAZE, and from 25.72/0.804 to 25.86/0.823 on O-HAZE. Compared with DNMGDT, the proposed method also achieves higher PSNR and SSIM on both real-world datasets. These results indicate that the proposed haze-aware modeling and feature enhancement mechanisms are better suited to complex real-world haze scenes, particularly in representing non-uniform haze distributions.

On the synthetic datasets, the proposed method also maintains strong performance. On SOTS-Indoor, it achieves a PSNR of 41.58 dB and an SSIM of 0.996, with the SSIM matching the best reported result. On SOTS-Outdoor, the proposed method obtains a PSNR of 37.88 dB and an SSIM of 0.991. Although these values are slightly lower than the best PSNR of 38.52 dB achieved by DNMGDT and the highest SSIM reported by some competing methods, they still place the proposed method among the leading state-of-the-art approaches. These results indicate that the proposed method enhances restoration performance in real-world non-uniform haze scenes while still maintaining strong adaptability to synthetic uniform haze scenes.

Furthermore, for the real-world non-uniform NH-HAZE dataset, the proposed method obtains the second highest PSNR and SSIM among the compared methods with available results, reaching 21.57 dB and 0.815, respectively. On the O-HAZE dataset, our method achieves the highest PSNR among the compared methods, with 25.86 dB, and obtains a competitive SSIM of 0.823. This result demonstrates its strong cross-dataset stability under different haze distributions, scene categories, and imaging conditions. These advantages mainly stem from two aspects. First, the haze-aware modulation mechanism can adaptively adjust feature responses according to the degree of local degradation, thereby enhancing the modeling of spatially non-uniform haze. Second, the deep feature enhancement module improves contextual modeling and feature reuse at the bottleneck layer, which helps recover structural information and texture details. In addition, the improved joint loss function further balances pixel reconstruction, structural consistency, and local contrast recovery, enabling the model to produce more stable dehazing results in complex real-world scenes.

In addition to restoration accuracy, computational complexity is also important for evaluating practical applicability. Therefore, we further compare the number of parameters and GFLOPs of representative dehazing methods, as shown in Table 3.

As shown in Table 3, DCAF-Net has a relatively higher computational cost than several lightweight dehazing methods. This is mainly because the proposed HDM and DFE modules introduce additional haze-aware modulation and gated dense feature reuse operations. Although these modules improve restoration performance under non-uniform haze and heavily degraded regions, they also increase the model complexity. Therefore, computational efficiency is not the main advantage of DCAF-Net, and lightweight architecture design will be further explored in future work.

In summary, the proposed method demonstrates strong overall performance in both synthetic uniform haze scenes and real-world complex haze scenes. In particular, it achieves superior results on NH-HAZE and O-HAZE, which validates the effectiveness of the proposed network in structure preservation, detail recovery, and cross-scene generalization.

#### 4.3.2. Qualitative Comparison

To evaluate the visual restoration quality of the proposed method, representative examples are selected from the SOTS-Outdoor, SOTS-Indoor, and O-HAZE datasets for qualitative comparison with DCP, FFA-Net, DeHamer, DEA-Net, and DenseDepth. Overall, all methods can alleviate haze-induced contrast degradation and detail blurring to different extents, but they differ in color restoration, texture recovery, depth preservation, and visual naturalness. In comparison, the proposed method removes haze while better preserving image structure and color consistency, and its restored results are closer to the reference images.

**Figure 4:**
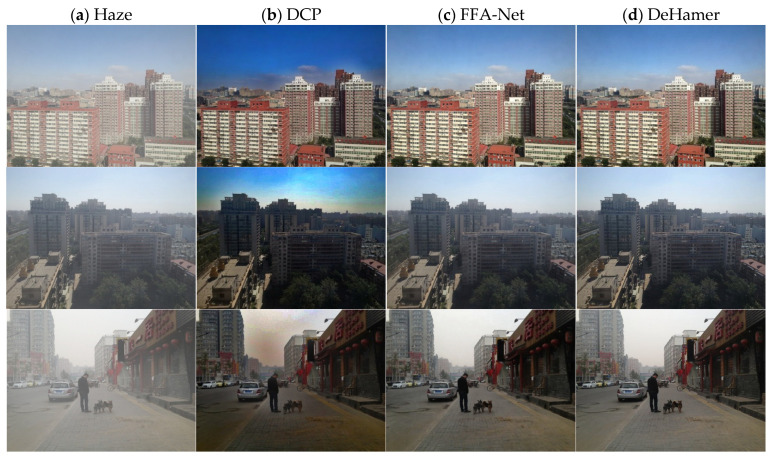

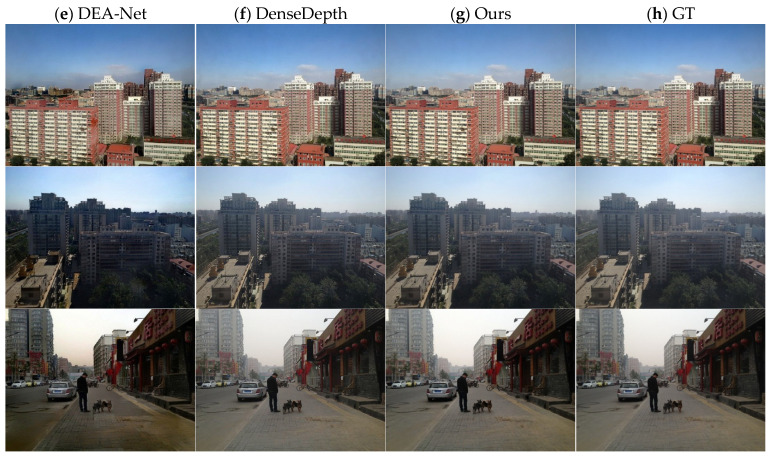
Visual comparison of the dehazing performance of different methods on the SOTS-Outdoor dataset.

As shown in Figure 4, in SOTS-Outdoor scenes, the images usually contain sky, buildings, roads, and distant regions, with evident depth variation. As a result, these scenes impose higher demands on distant restoration and color consistency. Although DCP can improve overall contrast, it tends to introduce color shifts or over-enhancement in sky and shadow regions. FFA-Net and DeHamer show good dehazing ability, but some local areas still suffer from insufficient detail recovery or uneven brightness. DEA-Net and DenseDepth can recover scene contours and major textures more effectively. By contrast, the proposed method performs more stably in distant building edges, road textures, and haze transition regions. It suppresses residual haze while avoiding excessive sharpening and color distortion, thereby achieving a better balance among clarity, depth perception, and visual naturalness.

**Figure 5:**
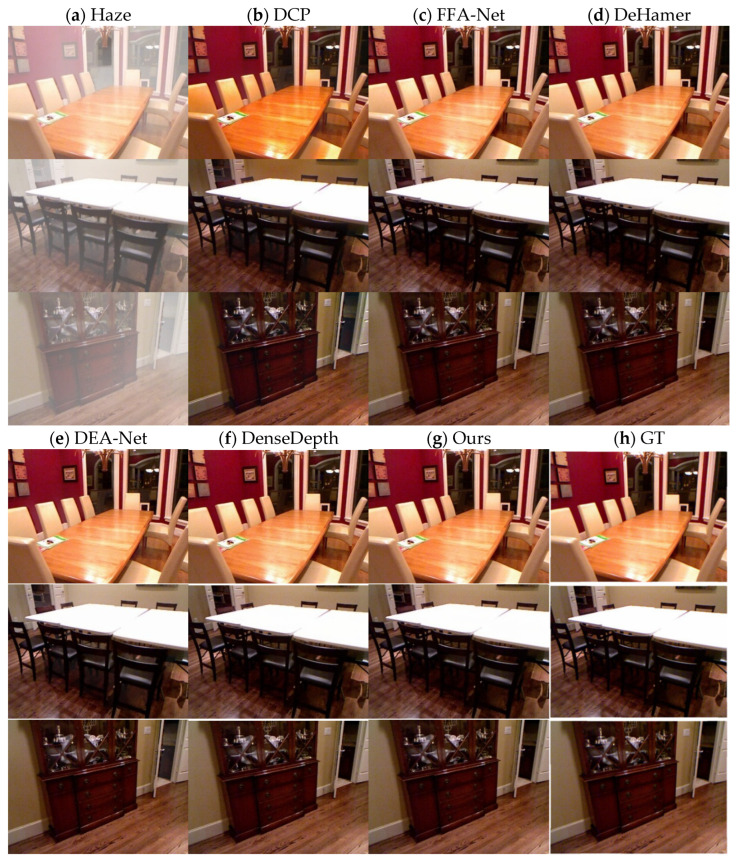
Visual comparison of the dehazing performance of different methods on the SOTS-Indoor dataset.

On the SOTS-Indoor dataset, the scenes mainly contain indoor objects such as tables, chairs, cabinets, and walls. As shown in Figure 5, greater emphasis is placed on color restoration, material detail recovery, and the preservation of structural boundaries. The visual results show that traditional methods usually struggle to balance brightness restoration and color stability in such scenes. For example, DCP tends to produce an overall warm color cast in some samples, and over-enhancement often appears in bright regions. In contrast, deep learning-based methods are generally more stable in indoor scenes and can better recover tabletop textures, chair contours, and the surface structures of wooden furniture. However, noticeable differences still exist among these methods. Some results appear visually clear, yet still suffer from overly high local color saturation, excessively sharp edges, or insufficient recovery of dark-region details. The proposed method demonstrates better color consistency and structural fidelity across multiple indoor examples. It restores details such as reflective tabletop regions, furniture edges, and cabinet textures in a more natural manner. Its outputs are also closer to the reference images in terms of overall brightness distribution and local contrast. These results indicate that the proposed method can effectively alleviate the low-visibility problem caused by dense indoor haze while maintaining strong stability in material texture and color restoration.

**Figure 6:**
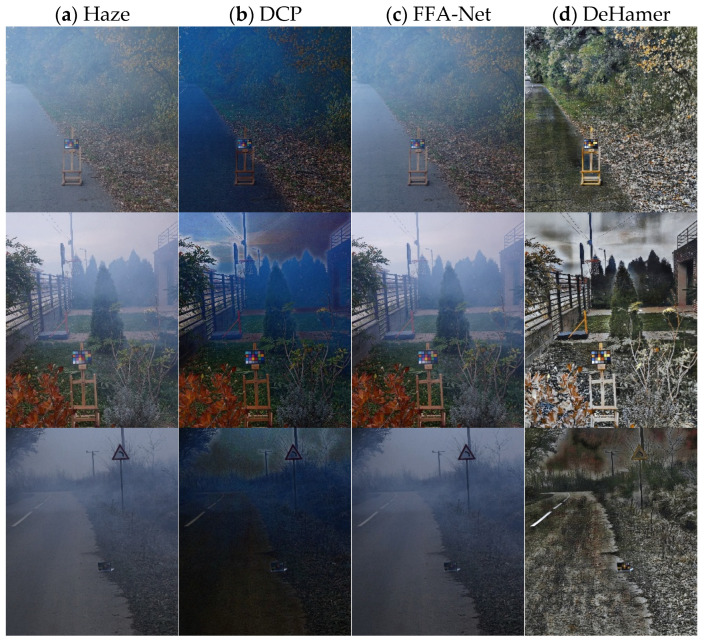

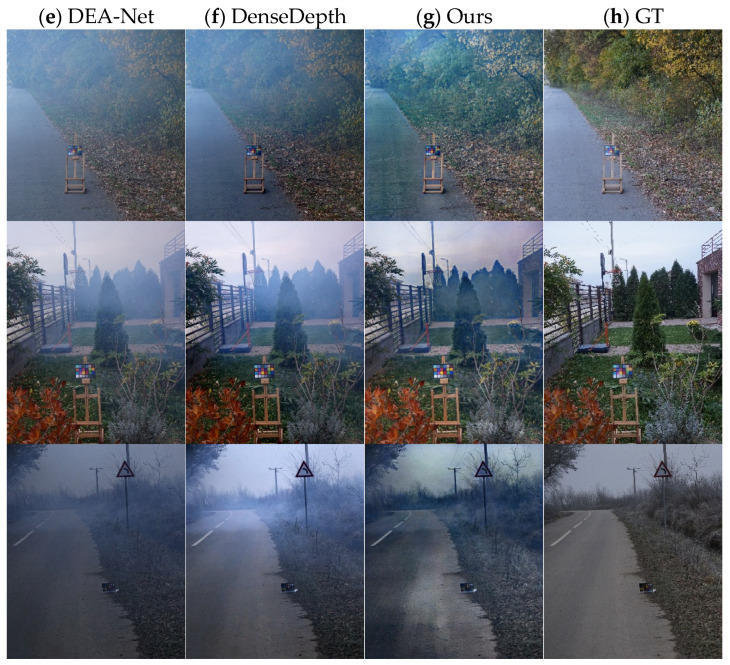
Visual comparison of the dehazing performance of different methods on the O-Haze dataset.

As shown in Figure 6, on the O-HAZE dataset, the images are captured from real haze scenes. As a result, the haze distribution is usually more complex and is often accompanied by non-uniform degradation, local illumination changes, and inconsistent blur levels between near and distant regions. Therefore, this dataset better reflects the generalization ability and robustness of a model in real application scenarios. The results show that under complex real haze conditions, the visual differences among methods become more pronounced. Although DCP improves image contrast in some examples, it also tends to introduce noticeable color deviations and dark-region distortion. This issue is especially evident in roads, trees, and distant background regions, where the restored results still differ substantially from the ground-truth images. FFA-Net performs relatively steadily in overall haze reduction, but it still leaves some residual haze in certain scenes, which makes distant regions appear less distinct. DeHamer shows strong local enhancement in some cases, but this may also lead to color imbalance or unnatural details. DEA-Net and DenseDepth can improve structural distinguishability in real hazy images to some extent, yet they may still suffer from residual haze, tone recovery errors, or insufficient texture restoration in local regions. In contrast, the proposed method produces smoother and more visually plausible restoration in regions such as trees, road edges, warning signs, and distant backgrounds. It more effectively alleviates the local contrast degradation and structural blurring caused by real non-uniform haze, while preserving the original color relationships and spatial depth as much as possible.

It should be noted that visual differences among recent high-performing methods on synthetic benchmarks are sometimes subtle and difficult to distinguish by global inspection. Moreover, local zoom-in visualization on low-resolution real-world images may introduce interpolation blur and may not reliably reflect the original texture quality. Therefore, the qualitative analysis mainly focuses on observable artifacts such as residual haze, color deviation, texture loss, and over-smoothing, while more detailed high-resolution local analysis and feature visualization will be explored in future work.

Taken together, the visual results on the three datasets show that the proposed method exhibits strong adaptability in both synthetic and real haze scenes. In outdoor scenes, it provides stable restoration for degraded distant regions and local structural boundaries. In indoor scenes, it achieves a good balance between color naturalness and material texture reconstruction. In real hazy images, even under more complex non-uniform haze distributions, the proposed method can still effectively suppress residual haze and recover major structural information. These observations are generally consistent with the quantitative results presented earlier. They indicate that the proposed network achieves a reasonable balance among detail recovery, structure preservation, and visual naturalness, and they further confirm its effectiveness under different scene conditions.

### 4.4. Ablation Study

To evaluate the effectiveness of each key component, this paper conducts ablation experiments on the O-Haze, SOTS-Outdoor, and NH-Haze dataset under the same training and testing settings. The baseline model is defined as the version without the haze-guided modulation module (HDM), the Deep Feature Enhancement module (DFE), and the mixed loss function (Mixed loss). Each component is then introduced progressively, and the results are reported in Table 4, Table 5 and Table 6.

Taking the ablation experiment on the O-Haze dataset as an example: as shown in Table 4, adding any single component improves the model performance. The baseline achieves a PSNR of 22.84 dB and an SSIM of 0.751. After introducing HDM, the performance increases to 23.73 dB and 0.797, indicating that this module enhances the model’s ability to perceive spatially non-uniform haze distributions. After introducing DFE, the model reaches 23.28 dB and 0.776, showing that deep feature enhancement helps improve the representation capability of the bottleneck layer. When the Mixed loss is adopted, the performance rises to 23.59 dB and 0.771, suggesting that the joint loss constraint improves the optimization balance between pixel reconstruction and structure preservation.

The results of two-component combinations further verify the complementarity among the proposed modules. Specifically, HDM + DFE, HDM + Mixed loss, and DFE + Mixed loss achieve PSNR/SSIM values of 24.99/0.798, 25.10/0.814, and 24.51/0.802, respectively. Among them, HDM + Mixed loss delivers the best performance, indicating a strong synergistic effect between haze-aware modulation and the joint loss constraint. By comparison, combining DFE with the other components also produces consistent improvements, which confirms its positive contribution to deep feature representation.

When HDM, DFE, and Mixed loss are all introduced, the complete model achieves the best performance, with a PSNR of 25.86 dB and an SSIM of 0.823. Compared with the baseline, this corresponds to improvements of 3.02 dB and 0.072, respectively. These results show that HDM, DFE, and Mixed loss enhance model performance from three complementary aspects: haze distribution modeling, deep feature enhancement, and optimization objective design. Their combination further improves the structural fidelity and detail restoration capability of the dehazing results.

The ablation results demonstrate the individual and joint contributions of the proposed components. HDM improves the adaptation to spatially varying haze-related degradation by providing explicit spatial guidance for feature modulation. DFE further enhances deep feature propagation and utilization, leading to improved recovery of heavily degraded regions and local details. The composite loss contributes to a better balance between reconstruction fidelity and perceptual quality. When these components are used together, the model achieves the best overall performance, indicating that HDM, DFE, and the composite loss function provide complementary contributions rather than isolated improvements.

### 4.5. Discussion

To further examine the behavior of DCAF-Net under related atmospheric degradation conditions, we conduct supplementary cross-degradation experiments on several image deraining datasets, including 200L, 200H, DDN, and DID.

As shown in Table 7, DCAF-Net achieves reasonable restoration results on the deraining datasets. Since the proposed model is designed for single-image dehazing rather than image deraining, these results should be interpreted as supplementary cross-degradation validation rather than direct comparison with specialized deraining methods. The results indicate that DCAF-Net can maintain a certain degree of robustness under related atmospheric degradation conditions, while dedicated multi-degradation restoration remains an important direction for future work.

To further clarify the methodological differences between DCAF-Net and representative recent dehazing approaches, Table 8 summarizes their main strategies, use of prior or density guidance, ability to model non-uniform haze, and main characteristics.

As shown in Table 8, most existing CNN-based and attention-based methods rely mainly on implicit feature learning or generic attention mechanisms to handle haze degradation. Transformer-based and diffusion-based methods improve global representation or generative restoration capability, but they may introduce additional computational cost and still often rely on learned implicit degradation modeling. In contrast, DCAF-Net explicitly incorporates complementary haze-related priors, including dark channel, local contrast, and saturation, to generate a haze density guidance map for adaptive feature modulation. In addition, the proposed DFE module improves deep feature reuse through gated fusion and residual propagation. These designs distinguish DCAF-Net from previous methods in terms of spatial degradation awareness and deep feature utilization.

## 5. Conclusions, Limitations, and Future Work

This paper proposes a Density-Conditioned Attention Fusion Network (DCAF-Net) for single-image dehazing, aiming to improve restoration under spatially non-uniform haze and complex degradation conditions. The proposed HDM integrates complementary haze-related priors, including dark channel, local contrast, and saturation, to generate a haze density guidance map for adaptive feature modulation and attention fusion. In addition, the DFE module enhances deep feature utilization through cross-layer feature reuse, gated fusion, and residual propagation. A joint loss function is further adopted to provide complementary supervision from pixel-level fidelity, feature-level perceptual representation, and structural consistency.

Experiments on synthetic and representative real-world hazy datasets show that DCAF-Net achieves competitive quantitative performance and favorable visual quality compared with the evaluated methods. The ablation results also verify the effectiveness of the proposed HDM, DFE, and joint loss function. These results suggest that introducing degradation-aware spatial guidance and enhanced deep feature reuse is beneficial for improving dehazing performance under non-uniform haze conditions.

Despite these advantages, several limitations remain. First, under extreme conditions such as very dense haze, low illumination, nighttime haze, sensor noise, or coupled multi-degradation scenarios, the model may still suffer from insufficient local detail recovery, residual degradation, or color deviation. Second, although HDM provides useful haze-related spatial guidance, the generated guidance map is not directly supervised by pixel-level haze density annotations, since such annotations are generally unavailable in existing dehazing datasets. Therefore, the map should be regarded as a degradation-aware guidance signal rather than an exact physical haze density estimation. In addition, although the selected priors in HDM are theoretically related to haze degradation, the current work does not provide a fine-grained quantitative analysis of each individual prior component. Future work will explore more explicit validation of haze guidance maps and more detailed prior-component analysis when reliable haze density annotations or physics-based references become available. Third, the evaluation on real-world hazy images is still limited by the scale and diversity of available paired real-world datasets such as O-HAZE and NH-HAZE. Although the results indicate promising restoration capability on representative real-world hazy scenes, they are not sufficient to establish comprehensive real-world generalization. More extensive validation on larger and more diverse real-world datasets, unseen domains, and mixed atmospheric degradation scenarios is needed. In addition, the current evaluation mainly relies on PSNR and SSIM, which may not fully reflect perceptual visual quality. Future work will incorporate additional perceptual quality metrics, no-reference image quality assessment, and user studies to provide a more comprehensive evaluation of restoration quality. Finally, the HDM and DFE modules introduce additional computational overhead in terms of parameters and FLOPs. This may limit real-time deployment on resource-constrained devices. Future work will therefore focus on lightweight architecture design, efficient attention mechanisms, model compression, and broader evaluation under complex open-world atmospheric conditions.

## Figures and Tables

**Table 1:** Detailed information on the datasets used in this study.

Datasets	Type	Training Images	Test Images
ITS	Synthetic	110,500	500
OTS	Synthetic	313,950	500
O-Haze	Real-World	35	5
NH-Haze	Real-World	45	5

**Table 2:** Quantitative comparison of image dehazing methods on multiple datasets. Bold indicates the best results, and underlining indicates the second-best results. ↑ indicates that higher values reflect better model performance.

Methods	Publication	SOTS-Outdoor		SOTS-Indoor		NH-HAZE		O-HAZE	
		PSNR ↑	SSIM ↑	PSNR ↑	SSIM ↑	PSNR ↑	SSIM ↑	PSNR ↑	SSIM ↑
DCP [2]	TPAMI’10	19.13	0.815	16.62	0.818	12.11	0.452	16.78	0.653
DehazeNet [6]	TIP’16	24.75	0.927	19.82	0.821	12.38	0.455	17.57	0.770
GridDehaze Net [23]	ICCV’19	30.86	0.982	32.16	0.984	18.19	0.608	23.48	0.726
MSBDN [7]	CVPR’20	33.48	0.982	33.79	0.984	19.35	0.640	24.36	0.749
FFA-Net [8]	AAAI’20	33.57	0.984	36.39	0.989	19.27	0.637	22.12	0.770
AECR-Net [37]	CVPR’21	-	-	37.17	0.990	19.46	0.641	23.21	0.749
DeHamer [24]	CVPR’22	35.18	0.986	36.63	0.988	20.66	0.680	25.11	0.777
FocalNet [38]	ICCV’23	37.71	0.995	40.82	**0.996**	20.43	0.790	25.50	**0.940**
DEA-Net [26]	TIP’24	36.03	0.989	40.20	0.993	20.20	0.762	22.84	0.751
DenseDepth [39]	PR’25	26.09	0.943	25.39	0.899	14.53	0.532	17.39	0.826
JSFC-Net [27]	AAAI’25	37.84	**0.994**	41.32	**0.996**	21.42	0.799	25.72	0.804
DNMGDT [31]	TMM’25	**38.52**	0.992	41.58	**0.996**	21.25	0.786	25.20	0.793
PGH^2^Net [32]	AAAI’25	37.52	0.989	**41.70**	**0.996**	-	-	25.47	0.880
BVIFormer [40]	Sci. Rep.’26	37.56	0.987	40.50	0.989	21.31	0.788	25.18	0.773
RPD-Diff [41]	BMVC’25	-	-	-	-	**23.44**	**0.820**	25.54	0.730
Ours	-	37.88	0.991	41.58	**0.996**	21.57	0.815	**25.86**	0.823

**Table 3:** Model complexity comparison of different dehazing methods.

Method	Params (M)	GFLOPs
DCP	-	9.48
DehazeNet	0.008	0.58
GridDehazeNet	0.958	21.40
MSBDN	31.35	41.54
FFA-Net	4.46	287.5
AECR-Net	2.611	52.20
DeHamer	132.4	48.93
DCAF-Net (Ours)	43.66	257.4

**Table 4:** Quantitative ablation study of the component modules in the DCAF-Net dehazing network on the O-HAZE dataset. (✓: module included; ×: module excluded)

Method	HDM	DFE	Mixed Loss	PSNR	SSIM
Baseline	×	×	×	22.84	0.751
Baseline + HDM	✓	×	×	23.73	0.797
Baseline + DFE	×	✓	×	23.28	0.776
Baseline + Mixed loss	×	×	✓	23.59	0.771
Baseline + HDM + DFE	✓	✓	×	24.99	0.798
Baseline + HDM + Mixed loss	✓	×	✓	25.10	0.814
Baseline + DFE + Mixed loss	×	✓	✓	24.51	0.802
DCAF-Net	✓	✓	✓	25.86	0.823

**Table 5:** Quantitative ablation study of the component modules in the DCAF-Net dehazing network on the SOTS-Outdoor dataset. (✓: module included; ×: module excluded)

Method	HDM	DFE	Mixed Loss	PSNR	SSIM
Baseline	×	×	×	36.03	0.989
Baseline + HDM	✓	×	×	36.72	0.990
Baseline + DFE	×	✓	×	36.25	0.989
Baseline + Mixed loss	×	×	✓	36.34	0.989
Baseline + HDM + DFE	✓	✓	×	37.12	0.991
Baseline + HDM + Mixed loss	✓	×	✓	36.89	0.990
Baseline + DFE + Mixed loss	×	✓	✓	36.91	0.991
DCAF-Net	✓	✓	✓	37.88	0.991

**Table 6:** Quantitative ablation study of the component modules in the DCAF-Net dehazing network on the NH-Haze dataset. (✓: module included; ×: module excluded)

Method	HDM	DFE	Mixed Loss	PSNR	SSIM
Baseline	×	×	×	20.20	0.762
Baseline + HDM	✓	×	×	20.83	0.793
Baseline + DFE	×	✓	×	20.67	0.781
Baseline + Mixed loss	×	×	✓	20.74	0.775
Baseline + HDM + DFE	✓	✓	×	21.09	0.809
Baseline + HDM + Mixed loss	✓	×	✓	21.11	0.803
Baseline + DFE + Mixed loss	×	✓	✓	20.88	0.794
DCAF-Net	✓	✓	✓	21.57	0.815

**Table 7:** Evaluation results on deraining datasets. ↑ indicates that higher values reflect better model performance.

Datasets	PSNR ↑	SSIM ↑
200L	37.54	0.973
200H	28.48	0.885
DDN	32.36	0.932
DID	33.51	0.941

**Table 8:** Comparative discussion table.

Method	Main Strategy	Prior/Density Guidance	Non-Uniform Haze Modeling	Main Characteristics
DehazeNet	CNN-based transmission estimation	Implicit haze-related features	Limited	Early learning-based method; depends on transmission estimation
GridDehazeNet	Multi-scale grid network with attention	No explicit density guidance	Implicit	Enhances cross-scale information exchange but lacks explicit haze distribution guidance
MSBDN	Multi-scale boosted dense network	No explicit density guidance	Implicit	Strong feature propagation but with relatively high complexity
FFA-Net	Feature attention network	No explicit density guidance	Implicit	Adaptively weights features but mainly learns degradation responses implicitly
DeHamer	Transformer-based dehazing	Haze-related priors/transmission-aware cues	Partially considered	Enhances long-range dependency modeling but may introduce additional complexity
DEA-Net	Detail-enhanced attention network	No explicit density guidance	Implicit	Improves detail preservation but lacks explicit spatial haze-density modulation
BVI-Former	Transformer-based restoration	No explicit density guidance	Implicit/learned	Uses Transformer representation for restoration; computationally demanding
RPD-Diff	Diffusion-based restoration	Degradation prior/diffusion process	Implicit/learned	Strong generative restoration ability but usually has high inference cost
DCAF-Net	Density-conditioned attention fusion network	Dark channel + local contrast + saturation guidance	Explicit spatial guidance	Uses complementary priors for haze density guidance and DFE for enhanced deep feature utilization

## Data Availability

You can access and obtain the data through the following links: ITS dataset: https://sites.google.com/view/reside-dehaze-datasets/reside-standard (accessed on 12 December 2017); OTS dataset: https://sites.google.com/view/reside-dehaze-datasets/reside-β (accessed on 12 December 2017); O-Haze dataset: https://data.vision.ee.ethz.ch/cvl/ntire18//o-haze/ (accessed on 13 April 2018); NH-Haze dataset: https://data.vision.ee.ethz.ch/cvl/ntire20/nh-haze/ (accessed on 7 May 2020).
